# Structured Total Knee Replacement Rehabilitation Programme and Quality of Life following Two Different Surgical Approaches - A Randomised Controlled Trial

**DOI:** 10.5704/MOJ.1907.004

**Published:** 2019-07

**Authors:** AP Antony-Leo, G Arun-Maiya, M Mohan-Kumar, PV Vijayaraghavan

**Affiliations:** Faculty of Physiotherapy, Sri Ramachandra Institute of Higher Education and Research, Chennai, India; * Department of Physiotherapy, Manipal Academy of Higher Education, Manipal, India; ** Department of Orthopaedics, Sri Ramachandra Institute of Higher Education and Research, Chennai, India

**Keywords:** arthroplasty, outcomes, physiotherapy care, medial parapatellar approach, mid-vastus approach

## Abstract

**Introduction:** The key important factor influencing the outcomes following rehabilitation is the surgical approach involved in Total Knee Replacement (TKR). Most studies have analysed the functional outcome in comparing the approaches on surgical perspective rather on post-operative therapeutic interventions. The current study was to analyse the effects of structured TKR rehabilitation programme on the quality of life and joint specific outcomes between two different surgical approaches.

**Materials and Methods:** In this double-blind randomised controlled trial, participants were randomly allocated to one of two groups: Group 1- those who underwent medial parapatellar approach and Group 2- those who underwent mid-vastus approach. Both groups received three-phase structured rehabilitation protocol for 12 weeks. The outcome measures of SF-36, knee mobility, isometric knee musculature strength and six-minute walk distance were measured at baseline, on discharge and at review after three months.

**Results:** The quality of life and joint specific outcome scores were better in mid-vastus approach than the popular medial parapatellar approach. The outcomes of knee flexion mobility (p=0.04), knee extension mobility (p=0.03), isometric muscle strength of quadriceps (p=0.001), isometric muscle strength of hamstrings (p=0.03), six-minute walk distance (p=0.001) and Physical Cumulative Scores (PCS) (p=0.03) were found to exhibit significant improvements at three months follow up.

**Conclusion:** The mid-vastus approach was found to exhibit better improvements following structured rehabilitation care, in physical summary scores of quality of life and joint specific outcomes than medial parapatellar approach.

## Introduction

Total knee replacement (TKR) is the most common, gold standard surgical intervention in relieving pain, improving physical functions and quality of life in end-stage osteoarthritis. Factors influencing the outcomes following rehabilitation after TKR include preoperative parameters, operative methods and postoperative care. A good outcome depends on the precision and accuracy of the prosthetic design, but is still further dependent on the surgical approach employed.

There are five commonly used TKR surgical approaches, namely, medial parapatellar, sub-vastus, mid-vastus, trivector and lateral parapatellar. The medial parapatellar approach is the gold standard and the most commonly used approach. Functional recovery of muscle strength and postoperative pain were noted to be better in mid-vastus than medial parapatellar approach had been reported.

The medial parapatellar approach is most commonly used for effective joint exposure. However, this approach stretches and compromises the integrity of the extensor mechanism^1^. The mid-vastus approach is a muscle sparing technique involving a midline incision and a deep dissection exposing the vastus medialis oblique (VMO) muscle.

The damage to VMO through the mid-vastus approach has been raised by researchers^2^. Thus, both these surgical approaches in TKR compromise the knee extensor mechanism, requiring specific post-operative therapeutic intervention to restore muscle function.

Studies^3-4^ concluded that the functional outcome as well as functional recovery of muscle strength and post-operative pain were noted to be better in mid-vastus than medial parapatellar approach. These studies have mostly analysed the functional outcome in comparing approaches on a surgical perspective rather on post-operative therapeutic interventions. Further, irrespective of the approaches, postoperative rehabilitation in these studies did not focus on the specific needs. Since muscle impairments are anticipated post-surgical complications following both surgical approaches, there is a need for a structured rehabilitation programme to mitigate them.

Previous studies have mainly analysed the functional outcome by comparing the effects of various surgical approaches rather than on the effects of post-surgical therapeutic interventions. Irrespective of the surgical approach, post-operative rehabilitation has not been focused on the need to remedy muscle impairment.

Hence the current study was undertaken with the primary objective of analysing the effects of structured TKR rehabilitation programme on the quality of life and joint specific outcomes between the two surgical approaches, namely the medial parapatellar and mid-vastus, in total knee replacement.

## Materials and Methods

The study was a double-blinded, randomised controlled trial, parallel group with balanced randomisation (1:1) conducted in Chennai, Tamil Nadu, India. The study was approved by the Institutional Ethics Committee (IEC- NI/11/APR/22/18). After a screening process, and giving a verbal explanation of the study protocol to the selected participants, informed consent was obtained from them.

The inclusion criterion for participants in the study were those who had undergone unilateral total knee replacement for degenerative joint disease of the knee joint, through medial parapatellar and mid-vastus surgical approaches, using posterior stabilised total knee prosthesis without patellar resurfacing. Patients awaiting revision TKR, post traumatic patients planned for TKR, and those with non-degenerative joint diseases, were excluded. The study was conducted in the physiotherapy department of participants referred from orthopaedic units of a tertiary care hospital in Chennai, Tamil Nadu, India, from September 2011 to December 2015.

The extent of the difference in six-minute walking distance test (6MWTD) between the two groups was estimated^5^. Using data obtained from a previous intense TKR rehabilitation trial, preliminary calculations determined that a sample size of 35 per group would provide 80% power to detect a 20% of difference in 6MWTD between groups at a significant level of p=0.05. Anticipating 10% loss to follow in each group, the sample size was calculated and rounded off to 80.

The process involved a screening of 150 possible participants, of whom, 105 fulfilled the eligibility criterion and were selected and enrolled into the study, and subjected to baseline evaluation and randomisation. The participants were randomly allocated using simple random sampling (allocation concealment method with the allocation ratio of 1:1), to either Group 1 (who underwent medial parapatellar approach) or Group 2 (who underwent mid-vastus approach).

The schedule of randomisation was prepared generated and monitored by an administrator in the outpatient orthopedic unit. The type of surgical approach (medial parapatellar and midvastus approach) was sequentially numbered and sealed in opaque envelopes. The allocation concealment mechanism was monitored attentively to prevent bias. The principal investigator randomly chose the envelope and rendered the postoperative care (Structured TKR rehabilitation).

The principal investigator, who was blinded of the group allocation, evaluated the baseline measurements and rendered postoperative structured TKR rehabilitation care to both groups. The outcomes were measured by an experienced physiotherapist who was blinded of the group allocation. The trial adhered to the procedures to maintain separation between the physiotherapist who rendered intervention and the physiotherapist who measured the outcomes. The outcomes were measured at baseline, on discharge (POST 1) and after three months.

The structured rehabilitation protocol^6^ was staged into three phases comprising early function phase (protective phase) from Day 1 to the 2nd week, progressive function phase (recovery phase) from the 3rd week to the 6th week and advanced function phase (activity phase) from the 7th week to 12th week.

Each phase comprised several categories including: prerequisite, goals, precautions, therapeutic exercise with frequency and duration, functional activities, criteria for progression and the outcomes measured. The progressive function phase and advanced function phase had sessions of mobility, stretching, strengthening, closed chain activities, balance training, functional training and aerobic conditioning. Phase 1 and part of phase 2 were considered inpatient phases followed by discharge planning, whereas part of phase 2 and phase 3 were considered home based rehabilitation under supervision.

All the participants in both groups were counselled about the TKR rehabilitation programme. Baseline measurements of primary outcomes and secondary outcomes were evaluated in the pre-operative period. Preoperative education sessions included pain management, role of therapeutic exercises, positioning and overall postoperative plan of care. All participants were provided with an educational booklet on TKR rehabilitation with pictorial representation of key exercises and tips for early recovery.

The Primary outcome tool - Quality of Life using SF-36 was obtained with permission from Quality metric (www.qualitymetric.com). The tool used was SF-36v2 four-week recall (license number-QM009932), certified scoring software v4.5 was used. The questionnaire had 8 domains namely, physical functioning (PF), role physical (RF), bodily pain (BP), general health (GH), vitality (VT), social functioning (SF), role emotional (RE) and mental health (MH). Both English and Tamil version of SF36 was used in this study. The short form health survey questionnaire (Tamil and English versions) was used to evaluate the health related quality of life.

The method of tool administration was self-administered, given to study participants in a printed paper format. The scoring software was used for summing responses and for transforming raw scores to 0-100, the higher the scores indicating better quality of life.

The secondary outcomes included knee mobility measurement of flexion and extension range using standard universal goniometry, isometric knee musculature testing using dynamometer and six-minute walk distance in meters.

The mobility of knee flexion and extension range was measured using standard goniometry^7^. To measure knee flexion mobility; the participants were positioned prone or side lying. The fulcrum of goniometry was placed over the lateral condyle of femur and the stationary arm was positioned over the long axis of femur and the movable arm parallel to the long axis of leg.

The isometric muscle strength of quadriceps muscle was tested with the subject positioned in high sitting with knee joint flexed in 70 degrees with the dynamometer probe placed over the lower end of the shin. Participants were instructed to push the dynamometer in attempting to knee extension. Each contraction of knee extension was held for five seconds and three trials were measured with thirty seconds rest interval between measurements. The same procedure was followed for measuring isometric strength testing of hamstring muscle except that the participants were positioned lying prone with available knee flexion. The mean values of three readings were calculated and considered as isometric muscle strength in kilograms.

The six-minute walk test is a functional ability test measuring the distance covered by a subject in six minutes. The procedure of the test was followed in accordance to the proposed guidelines of American Thoracic Society. The walking surface area was kept free of obstacles and two cones were placed at the two extremes.

Participants were instructed to discontinue walking, if any discomfort was felt and use of walking aid was optional. They were encouraged to walk back and forth the distance with normal speed and the distance covered in six minutes was measured in meters.

The data analysis of all outcomes were analysed for all randomised individuals using IBM.SPSS version (23.0). The normality of data was verified using Shapiro-Wilk test. Unpaired t-test was used to compare the groups for normal data. The statistical tests were considered significant, when the p value was less than 0.05.

## Results

**Fig. 1:**
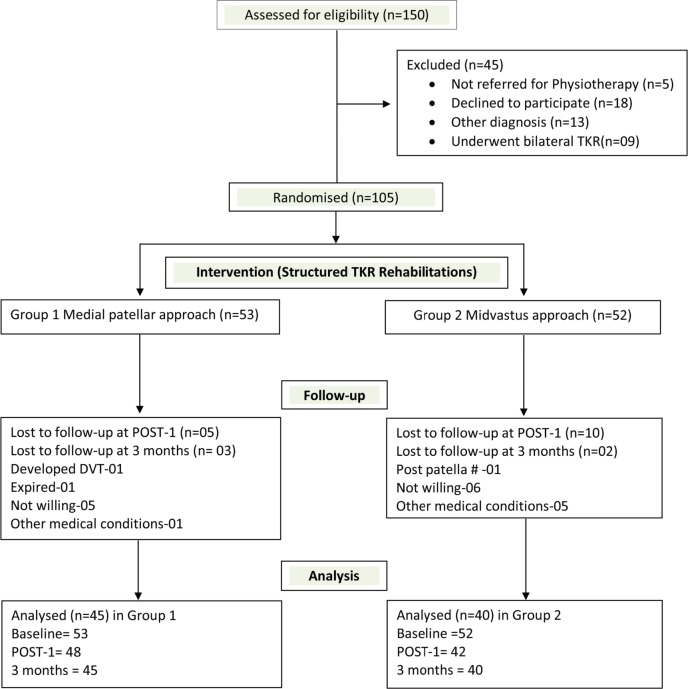
Consort flow chart.

A total of 105 participants were included in this study of which 85 participants completed follow up evaluation at three months. A total of 45 participants in Group 1 (medial parapatellar approach) and 40 participants in Group 2 (midvastus approach) completed the study with follow-up. The consort flow chart is illustrated in Fig. 1. The baseline demographics and clinical characteristics of both groups are shown (Table I). Primary (quality of life summary scores) and secondary outcomes compared between the two surgical approaches, namely medial parapatellar and mid-vastus approaches, using unpaired t test is summarised (Table II). Quality of life and joint specific outcome scores were better in mid-vastus approach than the gold standard medial parapatellar approach. The outcomes of knee mobility, isometric muscle strength of groups, six minutes walk distance and PCS scores were found to exhibit significant improvements at three months follow-up.

**Table I:** Baseline Demographics and Clinical Characteristics of both groups

Characteristics	MPP(n=45) Mean (SD)	MV(n=40) Mean (SD)
Age (years)	60.07(8.93)	60.23(8.08)
Height (cms)	154.46(9.46)	157.25(9.08)
Weight (kg)	70.06(14.44)	68.33(13.22)
		
BMI (kg/m^2^)	29.60(6.83)	27.74(5.42)
		
	**Numbers (%)**	**Numbers (%)**
Gender-Female	33(73%)	31(78%)
Gender-Male	12(27%)	09(22%)
Surgical side-Right	15(33%)	12(30%)
Surgical side-Left	30(67%)	28(70%)

**Table II:** Mean score changes of primary outcomes and quality of life summary scores between surgical approaches

	Outcomes	Mean (SD) MPP (n= 45)	Mean (SD) MV (n=40)	Mean diff.	p value
**Knee flexion mobility**				
FLEX-1	92.51(0.69)	92.25(1.11)	0.25	0.68
FLEX-2	82.43(1.33)	83.16(2.32)	0.72	0.56
FLEX-3	90.94(1.56)	94.32(2.94)	3.86	0.04*
**Knee extension mobility**				
EXT-1	5.86(0.65)	5.79(0.92)	0.07	0.89
EXT-2	5.94(0.87)	4.50(1.04)	1.44	0.04*
EXT-3	1.86(0.54)	0.90(0.51)	0.96	0.03*
**Knee flexor strength**				
FLXR-1	3.22(0.18)	3.34(0.29)	0.11	0.50
FLXR-2	3.11(0.19)	3.28(0.30)	0.16	0.34
FLXR-3	4.46(0.23)	4.92(0.37)	0.45	0.03*
**EXTR strength**				
EXTR-1	4.21(0.20)	4.59(0.30)	0.37	0.03*
EXTR-2	4.08(0.21)	4.68(0.38)	0.60	0.001*
EXTR-3	6.64(0.41)	7.69(0.61)	1.05	0.001*
**6MWT**				
6MWT-1	155.94(57.62)	160.69(61.65)	4.75	0.60
6MWT-2	119.5(77.40)	149.30(81.17)	29.78	0.01*
6MWT-3	271.60(135.98)	328.99(124.53)	57.38	0.001*
**PCS**				
PCS-1	39.63(1.57)	40.14(2.40)	0.50	0.72
PCS-3	48.36(1.16)	50.35(1.40)	1.99	0.03*
**MCS**				
MCS-1	45.30(1.70)	44.08(2.50)	1.21	0.42
MCS-3	51.98(1.61)	53.51(1.96)	1.53	0.23

FLEX-Range of knee flexion mobility in degrees; EXT- Range of knee extensor lag in degrees. EXTR-Isometric Quadriceps muscle strength in kilograms; FLXR-Isometric Hamstrings muscle strength in kilograms; 6MWT-Six minute walk distance in meters. PCS-Physical cumulative score; MCS-Mental cumulative score. * Significant at p< .05, unpaired t test

The maximum mean differences of knee extension mobility (1.44 degrees), isometric muscle strength of Quadriceps (1.05 kg) and 6MWT (57.38m) showed significant improvements in mid-vastus approach at discharge and at three months follow-up. Mental cumulative scores were equal and insignificant differences were noted between the two surgical approaches.

The most evident outcome at all the intervals of measurement favouring mid-vastus approach is the isometric strength of quadriceps muscle (7.69 kg) exhibiting 68% improvement from baseline values. Compared to medial parapatellar (68% improvement), mid-vastus approach exhibited early recovery of knee extension lag (88% improvement) and quadriceps isometric strength. This recovery was attributed to longer six minutes walk distance and improved physical cumulative scores (25% improvement) of quality of life in mid-vastus approach. The percentage of improvement in medial parapatellar group was 43% in six minutes walk distance and 18% in PCS.

The least percentage of improvement in both approaches was seen in the knee flexion mobility range (midvastus-2% improvement, medial parapatellar-2% decline). The isometric muscle strength of Hamstrings muscle showed 39% improvement (medial parapatellar) and 47% (midvastus) at three months from the baseline values.

Moreover, regaining of hamstring quadriceps ratio (midvastus-0.63, medial parapatellar-0.67) to normal state (0.60) was observed in both groups at three months. Hence, mid-vastus approach showed better improvements in joint specific outcomes and the physical component of quality of life as compared to medial parapatellar approach in TKR.

There were no adverse events reported following exercise training sessions. There were no events of fall or “near miss” during balance training sessions all throughout the programme. The loss to follow up was well within the limits, Group 1 (8 participants) and Group 2 (12 participants) and loss to follow-up were mostly observed following discharge from hospital. Medical co-morbidities were cited to be the reasons to loss to follow-up and two participants died due to hepatic complication and cardiac ailment.

All participants irrespective of groups received medical management for co-morbidities, post-surgical medication therapy and physiotherapy for a minimum of one month. None of the study participants received any other form of treatment or surgery for any associated disorders till final follow up. Almost all participants underwent consultations with the primary orthopaedic surgeon and physiotherapist at regular intervals.

## Discussion

The key finding of the study was that the validated structured rehabilitation programme for TKR was found to be applicable and safe to institute in patients undergoing primary TKR, irrespective of the surgical approach. The randomised controlled trial had attempted in all possible ways to increase participant’s compliance, the need for intent- to-treat analysis was not felt essential as the study has fulfilled the required sample size and the dropout rate was 25%. Despite attempts to improve adherence, the data of five participants were missed at three months follow-up.

Even though the study was able to meet the required sample size of 80, remaining missed data of five samples were used to analyse the impact of missing data in the conclusions. Following intent-to-treat analysis, there was not much difference in mean values of primary and secondary outcomes in both groups. Hence the study results discussed below are purely based on the achieved sample size of 85.

The contents of structured rehabilitation programme were supported in a systematic review of controlled trials recommending strengthening; intense functional exercise and balance training to be optimally included in outpatient physiotherapy protocol. Moreover the recommendation on application of FIT principles (dosage, frequency and duration) and criteria for progression of exercises was thoroughly implemented in the present study^8^.

The validated protocol was applicable from initial postoperative days and lasted till 12 weeks with greater application of therapeutics within initial four weeks following replacement surgery. Early starting of rehabilitation programme (within first postoperative month) was one of the factors for successful outcome in TKR^9^.

A similar RCT titled targeted rehabilitation to improve outcome (TRIO) after TKR was proposed^10^ based on the evidences of functional rehabilitation comprising range of motions, strengthening, proprioception and balance. Thus, the contents of structured protocol were in accordance to the recent evidences and practice and applicable for the two common surgical approaches in TKR.

Basic comparison between the two surgical approaches was analysed with early return of active straight leg raise (SLR) test and varied joint specific outcomes. The mid-vastus group showed early return of active SLR on the 6th postoperative day and the outcomes exhibited better improvements in mid-vastus approach than medial parapatellar approach^3^.

The stated surgical reason for early return of full SLR is due to the fewer lateral retinacular releases involved in midvastus approach^11^. This finding is in accordance with studies^1,4^ that mid-vastus is advantageous over the standard procedure in the early postoperative period. The key finding of significant improvements in isometric quadriceps muscle strength at POST 1 and at three months, further substantiates that mid-vastus is a muscle sparing approach in TKR procedure.

The Minimal Clinical Difference (MCD) observed in SF36 domains is 10 points^12^ and all domains reached the proposed MCD values in the mid-vastus group. In the medial parapatellar group, the domains of physical functioning and general health alone attained the proposed MCD score at three months but none of the mental domains reached it.

The percentage of change from baseline to three months in medial parapatellar group was 18% for PCS and 13% for MCS and overall PCS far bettered MCS. Whereas the midvastus group showed even improvements of change, PCS (20%) and MCS (18%) from its baseline cumulative scores. But a study^13^ revealed MCS bettered PCS but percentage of change from baseline to three months was 51% for PCS and 13% for MCS.

Overall, all domains improved significantly at three months when compared with baseline scores^14^. Baseline MCS scores of less than 42 indicated depressive disorders^15^ and predicted poor outcomes postoperatively with a specificity of 81% and sensitivity of 74%. The baseline scores of MCS in both groups were noted above 44, predicting better quality of life at follow-up.

The mean extension range (degree of extensor lag) and the physical summary scores (PCS) returned earliest in the midvastus group than the parapatellar group in the Indian population^11^. The significant differences of extension mobility (return of extensor lag) and improved isometric quadriceps muscle strength at two points of measurement were strong predictors of 6MWD in mid-vastus group with a mean difference of 57.38m in three months. The minimal detectable change (MDC) in knee joint extension mobility is 6.3 degrees following TKR was reported^16^. Our present study finding of 4.89 degrees was observed in mid-vastus group signifying the clinical progress in knee extension mobility over time.

The Hamstring-Quadriceps Ratio (H: Q) is a measure of importance in muscle function assessment. The ratio is considered to measure knee muscle balance and it is linked to reduce stresses on joint. The mismatch in the ratio is anticipated in preoperative stage and at early postoperative phase. The return of almost normal ratio in both groups highlights the balancing of knee musculature which was regained at three months following TKR.

Based on reviews of 6MWT, a study^13^ on progressive strengthening program exhibited 33% of change from its baseline values at three months. The current study showed 43% improvement (MPP group) as against 51% improvement in walking distance (MV group). Moreover, both groups showed clinically meaningful improvements in 6MWT as the proposed minimal clinical difference (MCD) value is 61.34m^17^. Based on the above stated discussions, the mid-vastus approach was superior to the standard parapatellar approach in terms of physical summary scores and certain joint specific outcomes.

The study was conducted in India using multiple outcome measures as per the International Classification of Functions. Apart from the key findings, the study was able to analyse return of muscle balance (Hamstring-Quadriceps Ratio) following knee replacement surgery.

The structured rehabilitation protocol has a component of balance training; outcome measure specific to balance skill (single leg stance time) was not implemented. Even though the exercise programmes are dosed in the structured rehabilitation program, there are chances of variations in exercise dosage in both groups. These findings were noted in a shorter duration of three months follow-up; however, a long term follow up of clinical outcomes between surgical approaches is highly warranted. All the above observations were considered to be the limitations of the current study.

## Conclusion

The mid-vastus approach was found to exhibit greater improvements in physical summary scores of quality of life and joint specific outcomes than compared to medial parapatellar approach following structured rehabilitation care. This study proved that mid-vastus approach was a muscle sparing approach facilitating early recovery, thereby achieving functional outcomes at three months following total knee replacement surgery in the Indian population.
